# Simultaneous, radiation-free registration of the dentoalveolar position and the face by combining 3D photography with a portable scanner and impression-taking

**DOI:** 10.1186/s13005-019-0212-x

**Published:** 2019-11-25

**Authors:** Lucas M. Ritschl, Klaus-Dietrich Wolff, Pia Erben, Florian D. Grill

**Affiliations:** Department of Oral and Maxillofacial Surgery, Technical University of Munich, School of Medicine, Klinikum rechts der Isar, Ismaningerstraße 22, 81675 Munich, Germany

**Keywords:** Dentoalveolar registration, 3D photography, Intra-extraoral registration

## Abstract

**Objectives:**

Simultaneous, radiation-free registration of the teeth and the upper and lower jaw positions in relation to the extraoral soft tissue could improve treatment planning and documentation. The purpose of this study is to describe a workflow to solve this form of registration and surface acquisition with a mobile device.

**Methods:**

Facial scans of ten healthy participants were taken using a blue-light LED 3D scanner (Artec® Space Spider; Artec® Group; Luxembourg). An impression of the maxillary dentoalveolar arch was taken simultaneously to the 3D photo using a modified impression tray with two different extraoral registration geometries (sphere vs. cross). Following, an impression of the mandibular dentoalveolar arch was taken once. Both impressions were scanned with the 3D scanner. All resulting standard tesselation language (.stl) files of the geometries were compared to the original, virtual .stl files and the root mean square errors (RMSE) were calculated for each surface (Artec Studio 13 Professional × 64; Artec® Group; Luxembourg) to determine which geometry serves as a better reference for intra-extraoral registration.

**Results:**

The RMSE between the original geometries and the scanned counterfeits were statistically lower for spherical geometries (*p* < 0.008). Once scanned and aligned, both geometries enabled an alignment of the intra- and extraoral scan. However, the spherical geometries showed virtually better results without significance (*p* = 0.70).

**Conclusions:**

The presented study provides a radiation-free solution for simultaneous dentoalveolar correlations in relation to the extraoral soft tissue. Spherical geometries achieved more precise and easier intra-extraoral alignments using the applied mobile 3D scanner and workflow.

## Introduction

The position of the maxillary dentulous or edentulous dentoalveolar arches in relation to the extraoral soft tissues is usually determined by using facebows and cast models that are positioned in an articulator after registration. To correlate the soft tissue and facial anatomy, auxiliary lines are marked on the models to transfer the patient’s situation as well and as realistically as possible [1]. This method, however, is susceptible to errors and may result in inaccuracies due to varying soft tissue situations, movements (e.g. grimacing), material properties in terms of shrinking and secondary deformation [2–4]. Three-dimensional (3D) photography is already used for various indications in dentistry and cranio-maxillofacial surgery, including esthetical dental rehabilitation of incisors, as a pre-interventional visualization tool to supplement the recorded information, treatment planning and follow-up documentation in orthognathic surgery [5–8]. This sort of mobile or stationary surface imaging is non-invasive and is becoming an additional gold standard tool for documentation and planning, especially in craniofacial surgery [9–12]. Several mobile systems have shown to be a valid and reliable solution with a reasonable cost–benefit ratio alongside the established expensive stationary systems of the last decade due to ongoing technical developments [11, 13, 14].

In terms of surface matching combining two different capturing methods, the combination of cone-beam computed tomography (CBCT) and 3D photogrammetry or scanned dental casts has proven to be a reliable and feasible method. An overview of various investigations was provided by Mangano and colleagues [15–17]. This results in good accuracy of the dental arch positioning and/or soft tissue illustration [18], which is necessary in pre-interventional planning of orthognathic surgery or orthodontic treatment and might facilitate the planning and simulation of a full mouth restoration. But of course, CBCT is associated with radiation and therefore should be restricted to defined indications with respect to the radiation protection law and current guidelines.

As a consequence, Bechtold et al. have described a radiation-free integration of a virtual maxillary dentoalveolar arch model into a facial scan in ten steps using a stationary photogrammetry system. This was found to have comparable precision to 3D-data derived from CBCT images alone [19]. In cases of an edentulous jaw Schweiger et al. as well as Hassan et al. presented a virtual workflow for complete dentures for which also facial scans were used. Their workflow aligns the digitalized dental arches according to the facial scan and provides valuable information to evaluate the tooth arrangements, however, without a definite intra-extraoral registration [20, 21].

The aim of this presented study was to analyse and describe a solution and workflow to register the intraoral position of the maxillary dentoalveolar arch simultaneously to the extraoral 3D photography with an intra-extraoral geometry using a portable 3D scanner. This would enable a virtual and radiation-free registration of the intraoral dental situation to the extraoral facial anatomy. The provided workflow could be used for prosthetic/orthodontic/orthognathic planning and post-interventional follow-ups and provides a recommendation for a straightforward geometry design and a step-by-step explanation.

## Materials and methods

### Applied software, hardware and analyses

**Fig. 1:**
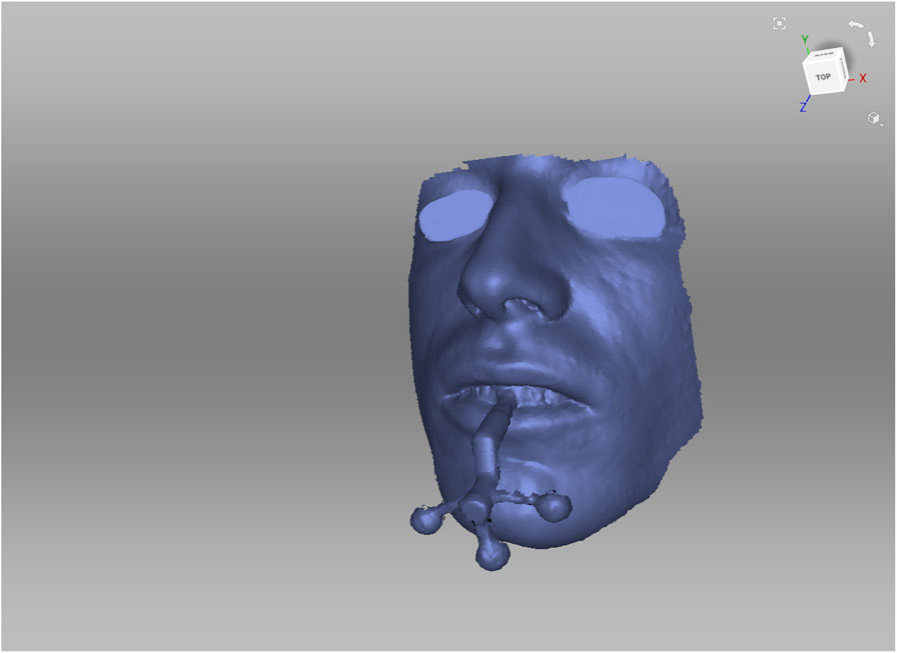
Standard tesselation language (.stl) file of one facial scan using a mobile 3D scanner. Simultaneous intraoral registration with a modified impression tray

**Fig. 2:**
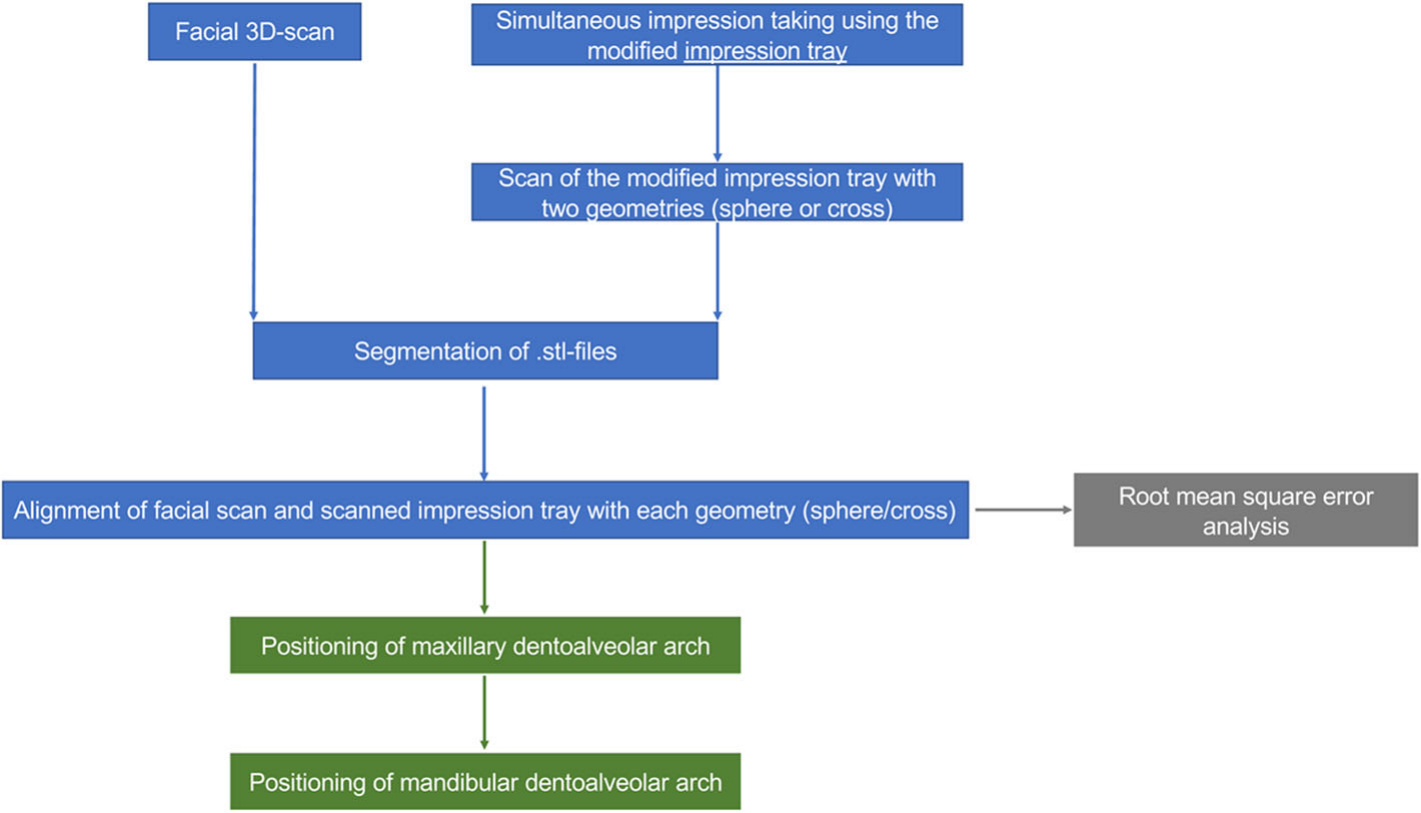
Applied workflow of face and geometry capturing and further analysis

**Fig. 3:**
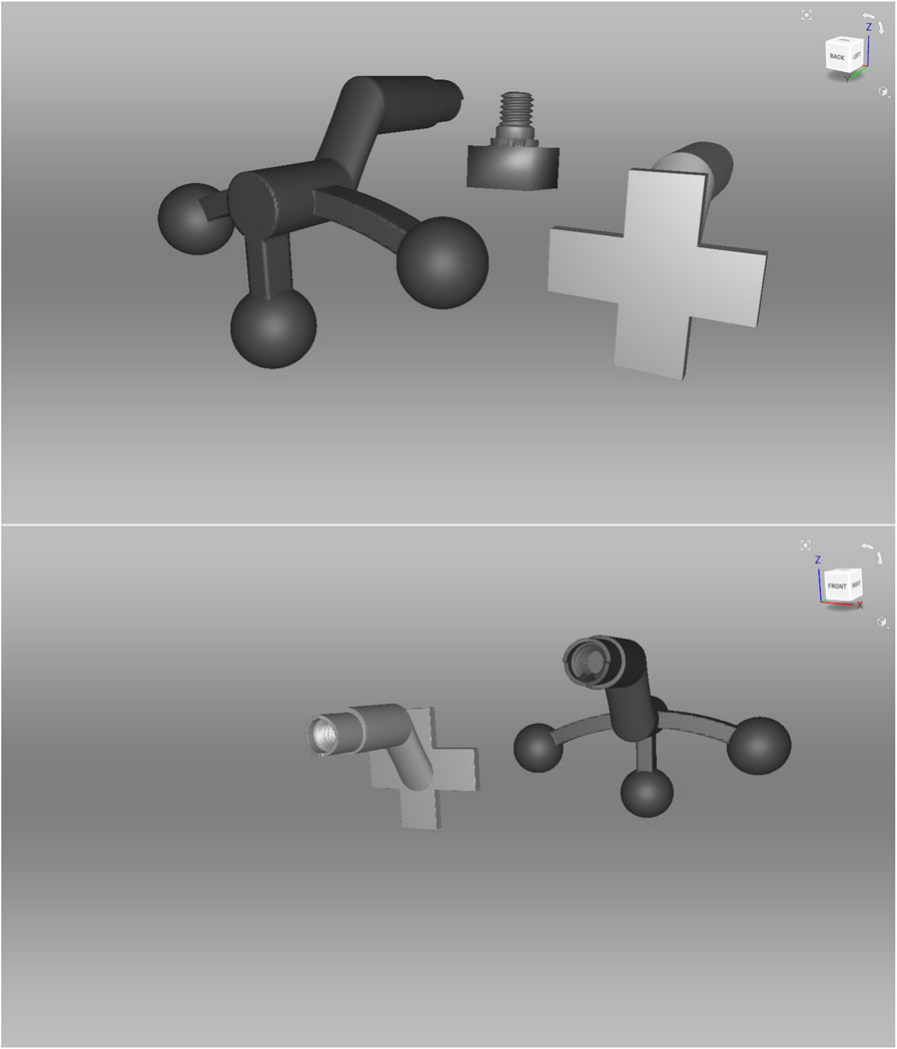
Extraoral Geometries. Top: Original standard tesselation language (.stl) file of the sphere (left), cross (right) geometries and threaded base (middle) in front side. Bottom: Corresponding back side view of both geometries

Facial scans of the enrolled participants were taken with a mobile blue-light LED 3D scanner (Artec® Space Spider; Artec® Group; Luxembourg). An impression of the maxillary dentoalveolar arch was taken simultaneously to the facial scan using a modified impression tray with two different extraoral registration geometries (sphere or cross) and A-silicon (Futar®D, Kettenbach Dental; Germany) (Fig. 1). As a preliminary investigation concerning the scanner used, we intended to evaluate the scannability of two kinds of extraoral geometries which were then compared: sphere vs. cross geometry (Fig. 2). These were adhesively attached to the threaded base of a common single-use plastic impression tray (Optitray®, Profimed, Germany) with an integrated screw. Further, an impression of the mandibular dentoalveolar arch was taken once. The threaded base and the corresponding screw within the extraoral geometries were designed virtually using common open-source CAD 3D software (Blender® Version 2.79; Blender Foundation and Institute; Amsterdam, Netherlands; and Meshmixer©; Autodesk Inc. Version 3.3) (Fig. 3). The geometries were printed in-house with the Form 2 stereolithographic printer (Form 2, Formlabs; USA) using a near-transparent resin (Clear Resin FLGPCL04; Formlabs; USA). The geometries were covered with a white ultra-thin CAD/CAM scan spray layer (HS CAD/CAM spray, Henry Schein® Dental; Germany) to enhance the visibility for the 3D scanner and to increase the accuracy of the captured geometries.

The mobile 3D scanner (Artec3D® Space Spider, Artec; Luxembourg) with a 3D resolution up to 0.1 mm and point accuracy up to 0.05 mm was used for capturing the facial surface and for digitalizing the impression-takings directly as described elsewhere [22]. To digitalize the dental plaster model, a 3D triangulation scanner (3Shape D500, 3Shape A/S, Denmark) was used.

Both impressions and the modified impression tray were scanned with the 3D scanner. All resulting standard tessellation language (.stl) files (dentoalveolar arches, sphere and cross geometry) were compared to the original, virtual .stl files of the digitalized plaster model. The root mean square errors (RMSE, [mm]) were calculated for each surface and aligned (Artec Studio 13 Professional × 64; Artec® Group; Luxembourg) to determine which geometry serves as a better reference for intra- and extraoral registration (Fig. 3) [11]. An analysis of the variance of a tenfold repetition of the digital workflow was performed.

### Workflow for simultaneous intra-extraoral registration in six steps

**Fig. 4:**
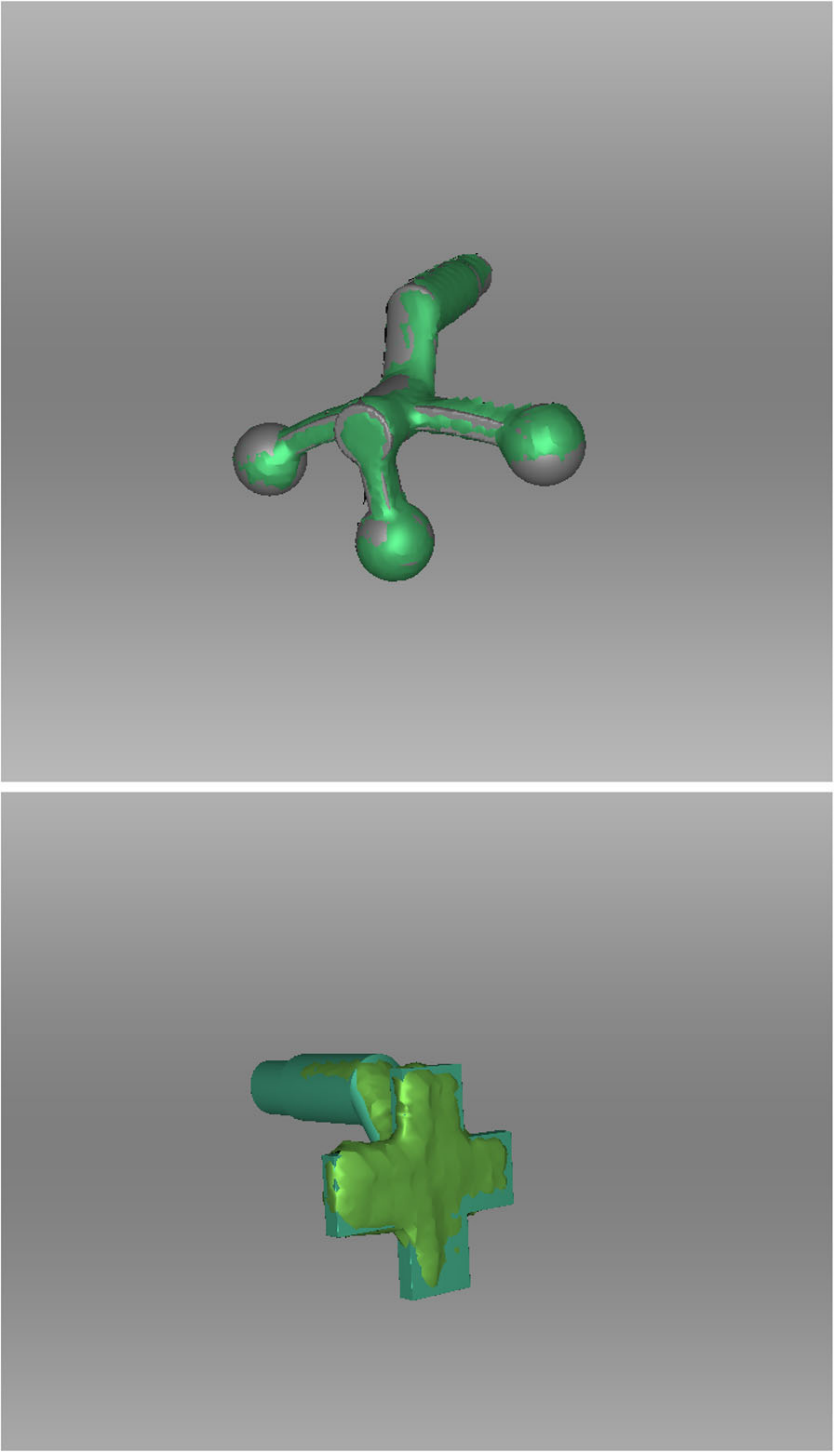
Alignment of the original .stl file and the resulting .stl file of the geometry after scanning. Top: Original (grey) and scanned (green) sphere file. Bottom: Original (dark green) and scanned (light green) cross file

All participants were scanned with both geometries in situ (step 1) and the geometries were scanned extraorally again (step 2). After segmentation and generation of corresponding .stl files of the 3D scans (step 3), the extraoral scans (Fig. 4) were virtually aligned using the Artec® Studio software with the scan of the impression tray by point selection in the geometries’ surfaces (Figs. 2 and 4) (step 4).

**Fig. 5:**
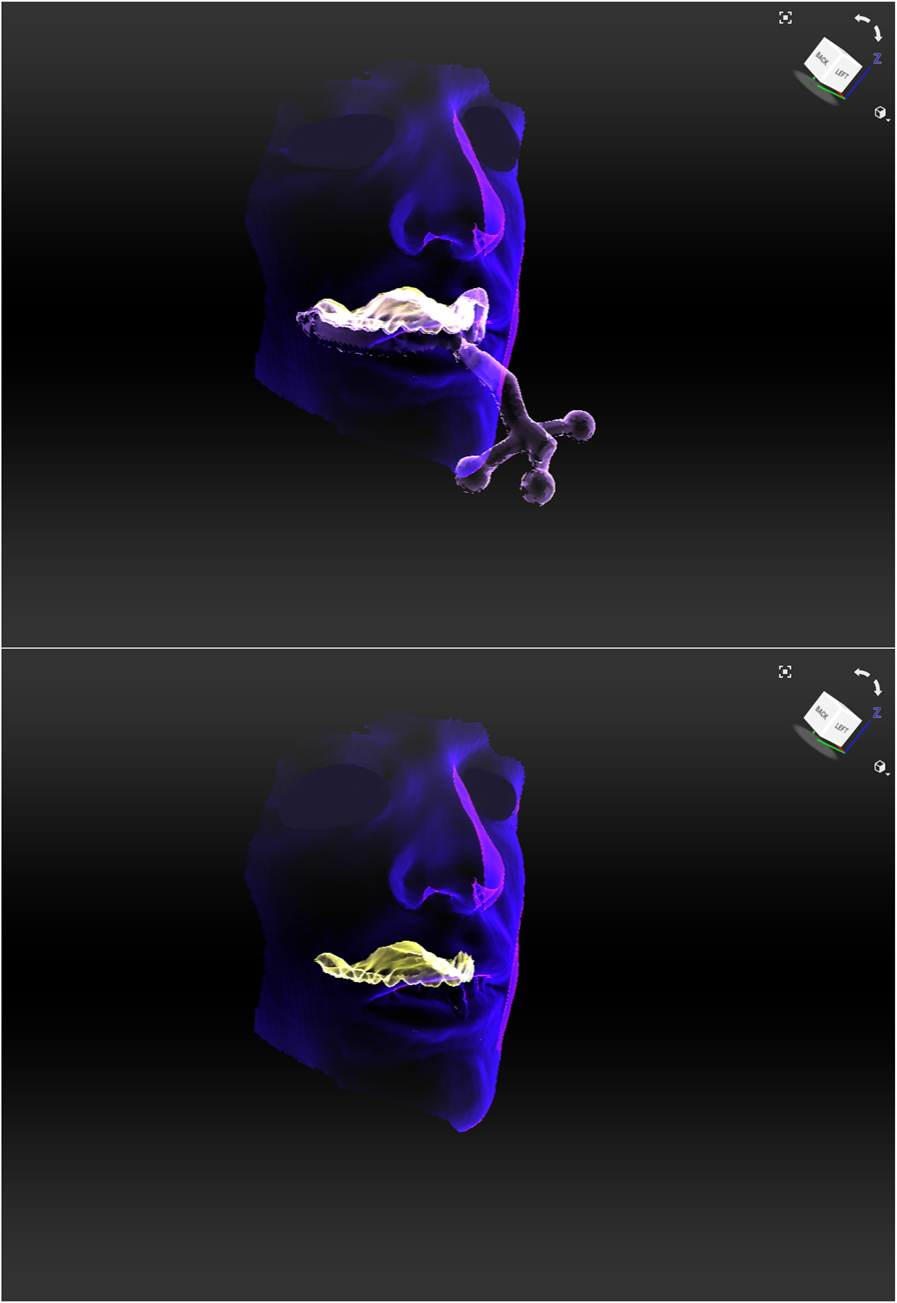
Alignment of the facial contours (blue) and the dentoalveolar impression. X-ray mode makes it possible to see the actual position of the dentoalveolar arch (yellow) in relation to facial contours

**Fig. 6:**
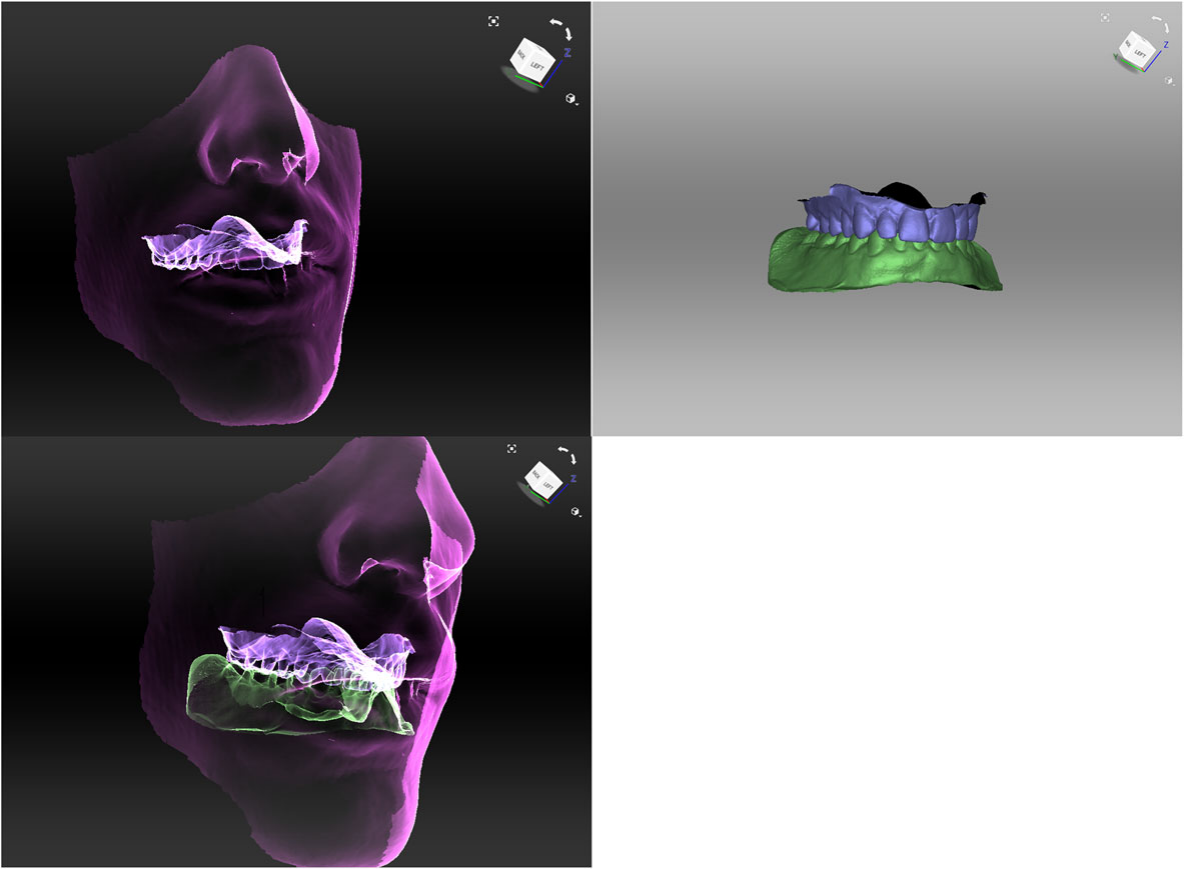
Facial scan in “X-ray mode” and after insertion of a virtualized 3D plaster model using the “normal inversion” to simulate the maxillary dentoalveolar arch position (top right). The mandibular dentoalveolar arch was positioned once according to the simultaneous registration (bottom left) after alignment of the virtually inverted maxillary impression model to the virtually inverted mandibular impression model (top left) using the occlusion points in maximal intercuspation

**Fig. 7:**
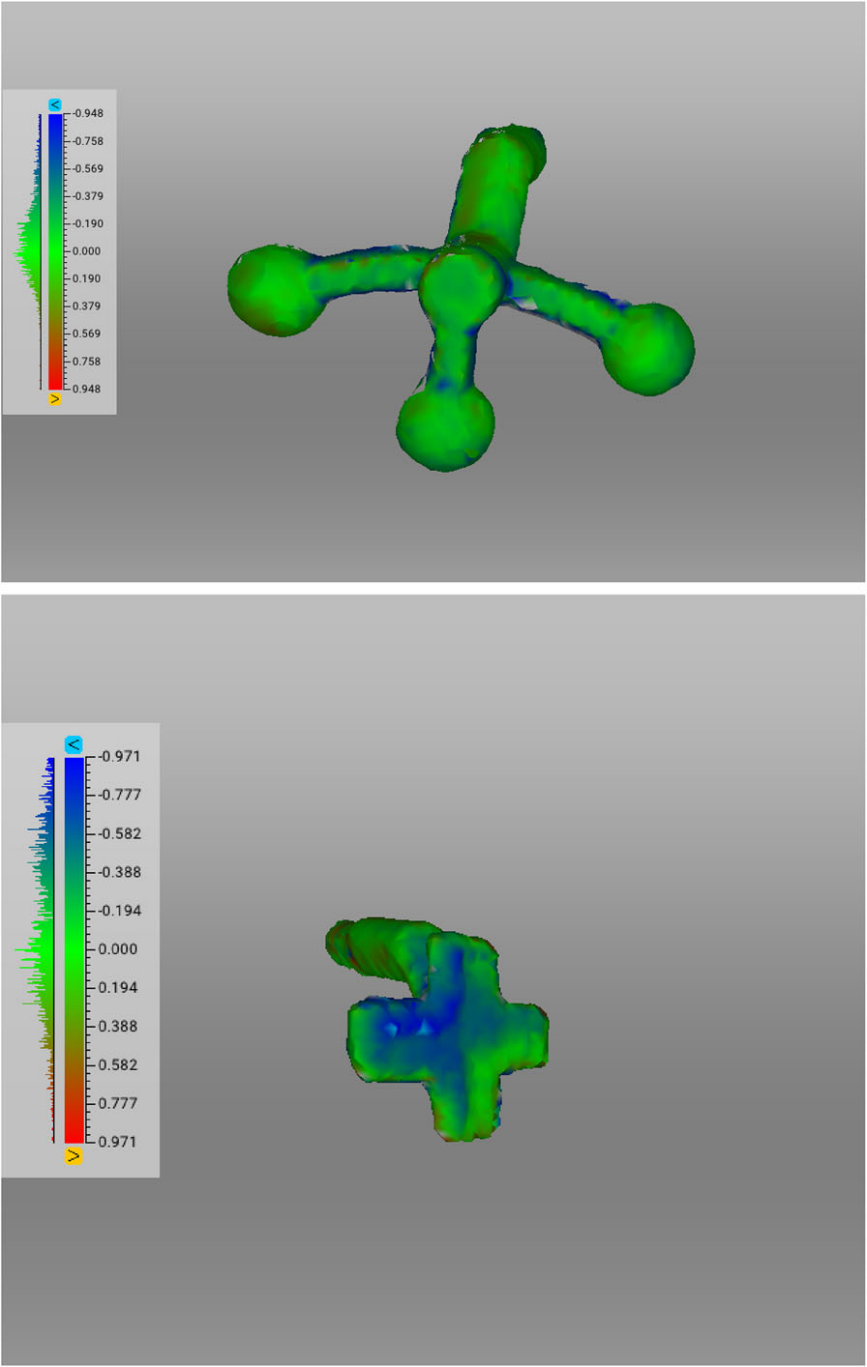
The root mean square error (RMSE) analysis [mm] of sphere and cross geometries after intra-extraoral alignment using the Software Artec® Studio 13 Professional (Artec Studio 13 Professional × 64; Artec® Group; Luxembourg)

This allowed an intra-extraoral registration of both scans (Fig. 5). Further, the impression was then segmented/separated from the rest, leaving only the impression of the dentoalveolar arch. With the function “normal inversion”, the impression of the dentoalveolar arches became the positive counterfeit (Fig. 6) (step 5). The facial scan was made transparent using the visual “X-ray mode” to facilitate the visualization of the position of the scanned maxillary structures. After alignments of the intraoral with the extraoral scans as well as the original physical geometries with their scans, an analysis calculating the surface deviations was undertaken represented by RMSE (Fig. 7). For further demonstration purposes a virtualized dental plaster model of a maxillary dental impression-taking were aligned to the scanned version along the gumline (Fig. 6). The corresponding mandibular dental impression was aligned along the occlusion points and also included in the 3D model (Fig. 6, step 6). The tenfold repetitions were performed additionally to gain information about the standard deviation and variance of RMSE. For this purpose, the workflow was repeated with the digitalized models starting from the above-mentioned step 3. After creating a data set as a basis for comparison, another ten repetitional data sets were formed. All ten data sets were then aligned individually to the basic data set with a consecutive RMSE analysis of all 3D models.

### Statistical methods

To represent surface deviations between two .stl files the RMSE was calculated. G-Power Software (Version 3.1) was used for the sample size calculation [23, 24]. For the evaluation of the scannability of the two geometries (cross vs. sphere) the RMSE of the original .stl file and the scanned counterfeits were compared and a sample size of five scans was calculated to be sufficient (Power: 0.95). Based on initially five scans comparing the RMSE analysis between the two kinds of scanned geometries after the virtual alignments, a sample size estimation resulted in eight necessary participants, which was extended to ten.

For analysis of differences the Wilcoxon signed-rank test was used. Statistical analyses were performed with the software R and its user interface R-Studio [25, 26].

## Results

**Fig. 8:**
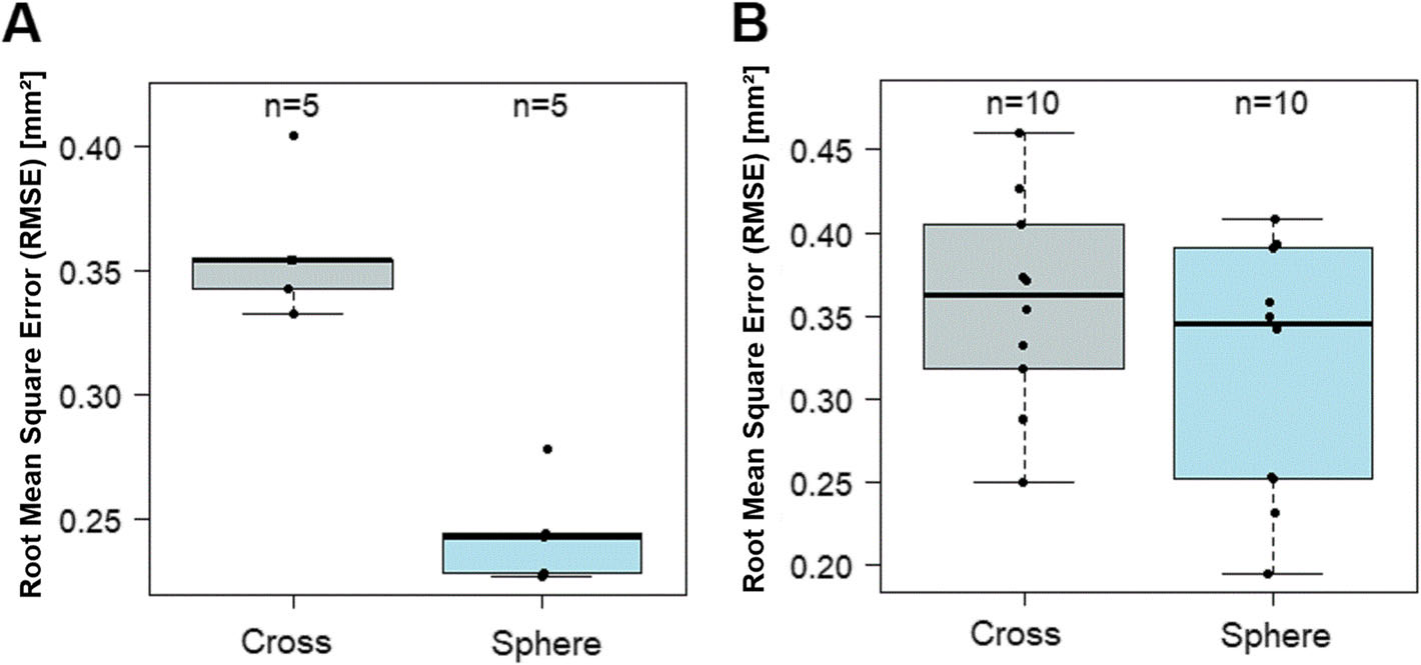
A: The root mean square error (RMSE) analysis [mm] comparing the alignments of the scanned cross (grey) and sphere (blue) with the original standard tesselation language (.stl) files alone (*n* = 5). B: RMSE analysis comparing the alignments of the cross (grey) with the sphere geometry (blue) within the facial scan (*n* = 10)

In a first step we analysed the accuracy of the alignments between the original, virtual .stl file and the scanned .stl file of the two geometries (cross vs. sphere) applying the RMSE analysis. The sphere geometries (*n* = 5; mean: 0.24 mm; range: 0.23–0.28 mm) showed significantly better results than the cross geometries (n = 5; mean: 0.36 mm; range: 0.33–0.40 mm; *p* < 0.008), (Fig. 7 and 8a, Table 1).

**Table 1:** Root mean square error (RMSE) analysis of the original standard tesselation language (.stl) files and the scanned counterfeits after virtual alignments (*n* = 5)

Participant	RMSE sphere geometry [mm]	RMSE cross geometry [mm]
1	0.23	0.33
2	0.28	0.34
3	0.23	0.35
4	0.24	0.35
5	0.24	0.40
Mean (range)	0.24 (0.23–0.28)	0.36 (0.33–0.40)
*p*-value	*p* < 0.008	

Ten healthy, Caucasian participants (four females and six males) with a mean age of 29.2 years (range: 27–32 years) were included in the clinical application and transfer. From all participants a facial scan was performed with simultaneous intraoral maxillary impression (Fig. 2). All impressions and scans were adequate in quality and could be used for further analyses. The two 3D files could be aligned digitally after extraoral digitalization of the impression tray in every case (Fig. 5). Once the geometries were scanned, there was no statistically significant difference in RMSE analysis between the cross and the sphere geometries (*p* = 0.70, Fig. 8b, Table 2).

**Table 2:** Root mean square error (RMSE) analysis of the *sphere* and *cross* geometries after alignments (*n* = 10) within the facial scan using a 3D scanner (Artec® Space Spider; Artec® Group; Luxembourg)

Participant	RMSE sphere geometry [mm]	RMSE cross geometry [mm]
1	0.39	0.33
2	0.19	0.46
3	0.25	0.43
4	0.23	0.35
5	0.35	0.37
6	0.39	0.37
7	0.25	0.25
8	0.36	0.29
9	0.41	0.41
10	0.34	0.32
Mean (range)	0.32 (0.19–0.41)	0.36 (0.25–0.46)
*p*-value	*p* = 0.70	

The consecutive exemplary alignment of a digitalized dental cast model along the gumline of the scanned impression and the positioning of the mandibular model along the occlusion points in maximal intercuspation was also possible in all cases, resulting in a complete virtual model indicating the three-dimensional position of upper and lower jaws in relation to the extraoral face (Fig. 6).

The tenfold repetition of the virtual alignment workflow showed a mean RMSE of 0.27 mm (range: 0.17–0.40 mm) with a standard deviation of 0.078 mm and a variance of 0.006 mm^2^.

## Discussion

Radiation-free solutions for intra-extraoral registrations are wanted in times of CAD/CAM-assisted surgery as well as increasing awareness and interest to health and radiation safety. Further, simultaneous registration and virtual and plaster-free workflows would reduce time and increase accuracy. Accuracy of facial plaster casts varies between 0.95 and 3.55 mm according to Holberg et al. [27]. This might be due to the reported finding that the influence of facial movements is greater than the technical influence in terms of technical error [28]. Grimacing is another common reason for insufficient quality for both direct 3D acquisition and indirect impression-taking as well as model or impression scanning [29, 30]. A quiet room with monotone walls and surroundings is therefore recommended for every kind of (3D) image-taking.

In addition, facial 3D photography has reached a high level of accuracy and reproducibility, even with portable devices [11, 13, 14]. Additionally, intraoral scanners have become a standardized and promising tool and the direct data capturing in terms of scanning/digitalization of the impression achieves more accurate results than the indirect/conventional way by creating a corresponding plaster model [22]. But a whole arch scan might be susceptible for more deviation in accuracy and should be restricted to ten units without wide edentulous areas [31, 32]. The direct scanning of dental arches takes longer than a conventional impression. Further, application is restricted to adults and to patients with regular mouth opening. The scanning time and the dimensions of the intraoral scanners are still too long and big for regular use in children or even newborns for diagnostic purposes or full virtual feeding-plate planning and production [33]. Therefore, our workflow for simultaneous, radiation-free intra-extraoral registration remains dependent on conventional impression-taking.

The idea of digital facebows that combine intra- and extraoral registrations using spherical geometries have been described and patented before [34]. Our geometries, which have been designed independently of the mentioned patent, have less contact to the lips and are in our opinion more easily transferred to the clinical setting. Bechtold et al. described a ten-step workflow for simultaneous intra-extraoral registration using a stationary photogrammetry system [19]. In contrast to their technique, our modified impression tray was much smaller and easier to design than their extraoral registration geometry and we only needed six steps for virtual segmentation and alignment. In contrast, we did not perform a control analysis of the maxillary or mandibular dentoalveolar arch position in correlation to the extraoral facial anatomy with a CBCT or comparable methodology after virtual alignment, something that is a common procedure in the literature [35]. There is no ethical approval granted by the Ethical Committee of the Technical University of Munich to perform a CBCT of our enrolled healthy participants. Therefore, this presented study focused on the accuracy of the two attached and scanned geometries as well as the feasibility of our virtual workflow and showed a low variance of alignments after a tenfold workflow repetition. The reduction of information when only performing six steps instead of ten seems to have only minor or even no impact. Here, the extraoral geometry showed best results in the RMSE analysis when the spherical geometry was used. This is in concordance to good results in the navigation-assisted surgery, where the intraoperative registration devices commonly also have spherical geometries for optimized tracking in the three-dimensional space. Spherical geometries can be detected from multiple angles easily [36]. We wanted to compare the standard geometry to the cross geometry, because automated registration and positioning of the geometry is wanted in a further step in our diagnostics and treatment planning for children with cleft lip and palate. A cross-like geometry has shown best results in this automated step (data not published) and would have been the missing link for fully automated generation of CAD/CAM-assisted appliances for nasoalveolar moulding (NAM) therapy as described earlier [37, 38]. Furthermore, a cross-like geometry seems to be more suitable for the alignment due to definite edges which can be used for reference marker positioning. However, our analysis showed that the spherical geometry is detected better by the scanner used in our clinical practice due to the technical scanning algorithm – the cross was also fully scanned but the edges seemed to be radiused. Since the scanner always needs a swing, e.g. for scanning the nose completely, the advantages of the detection of a spherical geometry compared to an edged geometry are pushed into the background. Once scanned, there were no statistically significant differences in RMSE analysis between the two kinds of geometries. For this purpose, we therefore need to perform more analysis on the basis of this feasibility study to improve the missing cornerstone. Next steps will be the design of individualized impression trays with an integrated threaded basis in order to abolish the need for an additional attachment of it to further optimize the CAD procedure.

Lin et al. and Jayaratne et al. compared the accuracy of low-dose cone beam CT scan protocols with the 3dMD system and obtained an RMS error between 0.74 ± 0.24 and 1.8 ± 0.4 mm [35, 39]. The precision of other stationary 3D camera systems is reported to be good, with the mean absolute differences for the VECTRA system lying within 1.2 mm and less than 1 mm by using 3dMD [40, 41]. These reported results are more precise than a deviation of 2 mm. RMS error values larger than 2 mm are considered unreliable according to the literature [11, 35]. Our tenfold repetition of alignment and the consecutive analysis of RMSE of the superimposed models showed a mean deviation of 0.27 mm with a standard deviation of 0.078 and a variance of 0.006. For documentation and illustration for the patient, this deviation is clinically negligible. Virtual surgery planning (VSP) is reported to be feasible, reliable and accurate. But nevertheless, the difference between the virtual plan and the postoperative result still ranges between 1 and 2 mm or up to ±12.5° in mandibular reconstructions using the free fibula flap and in VSP orthognathic surgery [42–45].

Nevertheless, studies comparing 3D photos compare only the “theoretical truth” with all the inaccuracies of the used systems [46]. Further, no technique enables a precise simulation and prediction of the postoperative result, yet. Within the reported and known limitations we therefore think that our results are clinically acceptable and relevant [47].

### Limitations

The study population may appear very small. But in a preliminary analysis of the expected accuracy of our geometry, this number with the corresponding power of 0.95 was calculated and granted for analysis by the Ethical Committee of the Technical University of Munich. We have not found the perfect geometry for automated registration and segmentation in the post-processing process. However, in this first feasibility study we wanted to define the best extraoral geometry for simultaneous intra-extraoral registration, with a small dimension that would be applicable in children as well.

In summary, the study presents an optimization of our chair-side 3D scanner which can be transported and used anywhere, in contrast to a stationary system. Despite having the advantage of being a hand-held device there are no cutbacks on a high scanning resolution as with other mobile devices such as tablets or smartphones. We show an easy-to-replicate six-step workflow that can be used for digital planning or pre- and postinterventional documentation which is intuitively accessible.

## Conclusion

Simultaneous, radiation-free intra-extraoral registration is possible and we described a six-step approach to solving this interesting and promising procedure, which can be applied in many fields in modern documentation and treatment planning. Our results implied a superiority of spherical geometry for extraoral registration.

### Clinical significance

Our analysed workflow for simultaneous dentoalveolar and extraoral soft tissue registration enables a radiation-free solution and can be applied in many fields of treatment planning and documentation.

## Data Availability

The data used for analysis has been referenced in the text or tables of the paper.
